# Total mercury contamination in fish species of Northwestern Ecuador and potential human health risks

**DOI:** 10.1371/journal.pone.0342455

**Published:** 2026-02-19

**Authors:** Gabriela S. Yánez-Jácome, Andrés Merino-Viteri, Eduardo Rebolledo Monsalve, Roberto Xavier Supe Tulcan, Laurence Maurice, Hugo Navarrete

**Affiliations:** 1 Centro de Estudios Aplicados en Química (CESAQ), Pontificia Universidad Católica del Ecuador, Quito, Ecuador; 2 Laboratorio de Ecofisiología, Facultad de Ciencias Exactas, Naturales y Ambientales, Pontificia Universidad Católica del Ecuador, Quito, Ecuador; 3 Escuela de Gestión Ambiental, Pontificia Universidad Católica del Ecuador Sede Esmeraldas, Esmeraldas, Ecuador; 4 Laboratory for Earth Surface Processes, Ministry of Education, College of Urban and Environmental Sciences, Peking University, Beijing, China; 5 OMP-GET laboratory, Univesité de Toulouse-IRD-CNRS-CNES, Toulouse, France; 6 Facultad de Ciencias Exactas, Naturales y Ambientales, Pontificia Universidad Católica del Ecuador, Quito, Ecuador; King Faisal Specialist Hospital and Research Center, SAUDI ARABIA

## Abstract

Gold mining activities are often suspected to increase mercury pollution-associated with human health and ecological risks in aquatic ecosystems. The objective of this study was to quantify total mercury (THg) concentrations in fish from four different sampling sectors along the Cayapas River watershed that varied according to different exposure levels to artisanal and small-scale gold mining (ASGM) activities. We analyzed 142 samples from eight freshwater fish species (*Bryconamericus dahli*, *Brycon* sp.*, Brycon dentex, Chaetostoma marginatum*, *Pimelodella modestus*, *Rhamdia quelen*, *Gobiomorus maculatus*, and *Mesoheros festae*). Potential human exposure and health risks from fish consumption in three population groups (children, women, and men) was also evaluated to estimate intake rates and determine fish consumption with minimal risk to the population’s health. We found different THg concentrations among feeding habits and sampling sectors. Carnivorous fish species (*Pimelodella modestus*, *Rhamdia quelen*, *Gobiomorus maculatus*, and *Mesoheros festae*) showed higher THg concentrations (0.063 ± 0.021 µg.g^-1^) and periphyton-feeder species (*Chaetostoma marginatum*) revealed the lowest levels (0.018 ± 0.007 µg.g^-1^). Downstream sites showed the highest levels of THg compared to the other upstream sites, despite some sites being directly impacted by ASGM activities. Regarding human exposure, no significant potential health risk was found for the exposed population over a lifetime. However, the THg of a *Rhamdia quelen* sample slightly exceeded the FDA-EPA Hg reference value in fish across all sites, representing a potential risk for children. Our results suggest that the THg concentrations in the studied fish species are independent of ASGM activities. The accumulation rates may be due to other parameters such as land uses, local hydrology, fishing pressure or natural habitats modification. Further ecological and physiological studies, including spatial and seasonal distribution of Hg in the surface sediment, water column and fish species, should be investigated to assess and modulate the impacts of the ASGM in the Santiago-Cayapas watershed compared with other land uses that contribute to the Hg inputs, bioaccumulation, and biomagnification in ichthyofauna.

## Introduction

Mercury (Hg), a global contaminant, is a toxic trace metal originated from both natural and anthropogenic processes [1,2]. As Hg changes among its different chemical forms [3,4], it can be transported and recycled between major environmental systems and enter food chains, potentially affecting organisms [5]. Several anthropogenic activities including improper disposal of consumer products (e.g., batteries, fluorescent light bulbs), medical waste incinerators, paper production, smelting of non-ferrous metals, iron and steel foundries, the cement industry, excessive use of inorganic fertilizers, among others, increase the global Hg emissions into the environment [4–11].

Mercury is released into aquatic ecosystems where elemental and inorganic forms are predominant [12,13]. In aquatic environments, inorganic mercury is methylated through biological and non-biological processes to methylmercury (MeHg) [14,15] —a potent neurotoxin and the most toxic form of Hg [16–18]. When elemental and inorganic Hg reaches the water bodies, it can be deposited in the bottom sediments or bound to suspended particulate matter. It also can be oxidized into inorganic Hg salts (Hg^+^, Hg^+2^) and then converted to MeHg [19–21]. Hg concentrations can be much higher in aquatic organisms than their surrounding waterbodies due to the combination of bioaccumulation and biomagnification [22,23]. Since fish have a higher capacity to accumulate Hg than invertebrates [24] and are easily sampled [25], some fish species (especially sedimentary ones) are useful bioindicators of the levels of pollution of aquatic ecosystems [24,26,27].

Broad-scale changes to the landscape and habitat use have been linked to high levels and bioaccumulation of Hg in organisms [28]. Soil disturbances because of deforestation, slash-and-burn agriculture and/or cattle raising, following human colonization and landscape modifications, increase erosion of soils, mainly during rainy seasons, and enhance the release of Hg, allowing it to enter waterbodies affecting the bioaccumulation in the aquatic chain [29–35].

Artisanal and small-scale gold mining (ASGM) is the largest anthropogenic contributor of Hg emissions into the atmosphere, with 1,400–2,200 tons per year [36]. It is also the largest global source of freshwater Hg releases [37] mainly related to the indirect soil erosion from deforestation associated with mining operations. For example, in Amapá, Brazil, deforestation caused by buffalo ranching, agriculture, ASGM, and population growth have contributed to increased soil erosion and particulate and colloidal transport of Hg into the aquatic ecosystem [38]. Even more concerning, Diringer et al. (2020) [39] reported that in Peru’s Region of Madre de Dios, deforestation from ASGM has exacerbated Hg transport and carried anthropogenic and geogenic Hg from land surfaces to water bodies, resulting in major socioeconomic shifts in the region and national state of emergency in response to concerns for wide-scale mercury poisoning by ASGM. Mainville et al. 2006 [40] demonstrated that in the Ecuadorian Amazon, elevated deforestation rates and the proximity of volcanoes could have played an important role in soil erosion. These factors were suggested to have exposed the layer with high Hg burden, accelerated Hg leaching and contributed to the fish Hg contamination in the Napo River watershed.

In South America, ASGM represents 40.6% (340 tons) of the total global Hg burden, followed by Sub-Saharan Africa with 30.1% (252 tons) [37]. ASGM activities not only cause widespread deforestation but also create mining waste which is discharged directly to soils and rivers [41], negatively affecting the environment [42,43], tropical forests [44], aquatic and terrestrial biodiversity [45], and riverside communities located around and downstream of gold mining areas [35,46–48].

Illegal ASGM has been practiced in Esmeraldas province (Ecuador) since 2005, even though mining activities are forbidden in this area since March 2011 [49]. An injunction issued by a judge prohibited mining exploitation, whether legal or illegal, in the San Lorenzo and Eloy Alfaro counties due to the use of machinery and Hg in the open air [50]. In 2011, an environmental monitoring assessment was performed in Eloy Alfaro and San Lorenzo, analyzing the quality of water bodies. Mercury and other trace metals were measured in water, sediment, fish, crustaceans, and mollusks collected in rivers Bogotá and Santiago, and their affluents Tululbí, Cachabí, Palaví, Estero María, and Zapallito showing possible effects on fish, and on the loss of flora [49].

Rebolledo Monsalve et al. 2022 [51] studied water quality and fish communities of rivers and streams in the Santiago-Cayapas watershed, located in an area affected by ASGM. It was found that open-pit mining causes a reduction of dissolved oxygen concentrations and an increase of water temperature, turbidity, and metal concentrations (aluminum, chrome, cobalt, copper, iron, manganese and vanadium). Additionally, fish abundance decreased in streams that drain active mines, showing that the response of fish communities to open-pit mining depends on the pollution tolerance of each fish species and the presence of speciﬁc adaptations to turbid waters [51]. However, few studies related to THg content in fish have been conducted in the Northern Esmeraldas which is unfortunate, as they constitute an important part of the local population’s diet.

In contrast to previous studies, this study aims to identify the impact of ASGM on Hg concentrations in fish in the Northern Esmeraldas province relative to other parameters. THg concentration was determined in fish muscle caught in ten rivers from the Santiago-Cayapas Basin (Comba, Quebrada del Parto, Cachabí, San Javier, Tululbí, Durango, Uimbicito, Estero María, Zapallito, and Santiago). The aim was to compare the impact from the ASGM (sampling sector), fish size, dietary habits, and geographic location of the sampling sites relative to the Cayapas River mouth. Additionally, we evaluated the potential human health risk from regular to extreme consumption of freshwater fish, in the same study area.

## Materials and methods

### Study area and fish sampling

Fish samples were collected in 2019 during the end of the dry season (December), in the Santiago-Cayapas watershed, located in the Northern of the Esmeraldas province, Ecuador. The Santiago-Cayapas watershed is the third largest hydrological basin (6,321 km^2^) draining to the Pacific Ocean in Ecuador. Annual rainfalls average 3,326 mm, so basin water levels are high throughout the year [52] but discharge seasonally, with higher flows between January and April.

**Fig 1:**
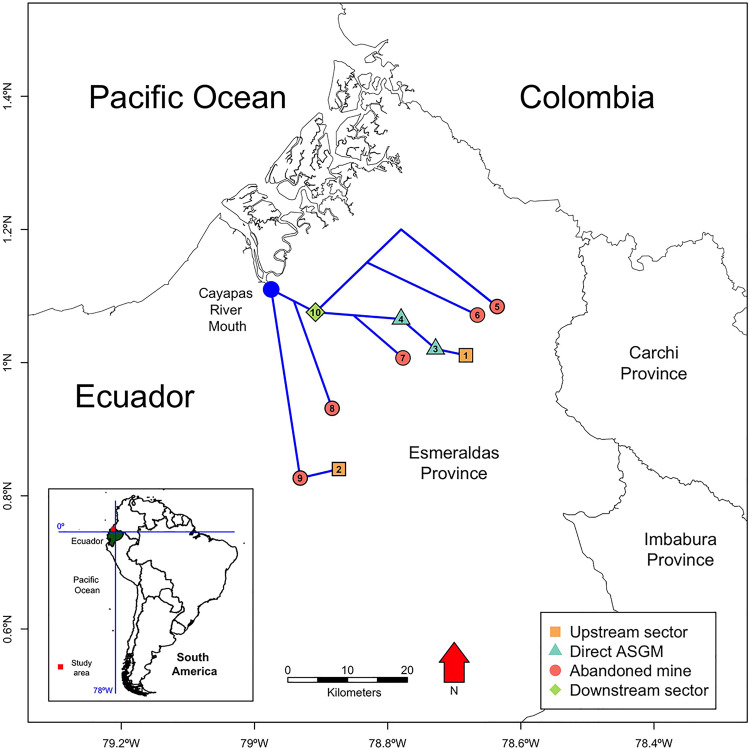
Schematic distribution of fish sampling sites along the Santiago-Cayapas watershed, Esmeraldas province, Ecuador. Sites are classified according to their potential influence of ASGM: upstream sector (1. Comba, 2. Quebrada del Parto), direct (3. Cachabí, 4. San Javier) or abandoned mining activities (5. Tululbí, 6. Durango, 7. Uimbicito, 8. Estero María, 9. Zapallito), and downstream sector (10. Santiago). The map was assembled using different open sources [53].

Sampling sites were characterized into four different sectors relative to their exposure to ASGM activities (Fig 1). These sectors included: a) upstream, potentially unaffected sites (Comba and Quebrada del Parto); b) direct ASGM sites in rivers that were influenced by mining (Cachabí and San Javier); c) abandoned gold-mine sites that have not had mining influence more than six months ago (Tululbí, Durango, Uimbicito, and Estero María); d) downstream site (Santiago) watershed that receives water from tributaries with and without mining influence. These categories were considered as qualitative variables for further statistical analysis.

Airline distance was calculated from each sampling site to the Cayapas River mouth (79.0°W, 1.16°N) using the function *LineLength* from the sp package [54] in R software [55], based on an Ecuador´s elevation raster with a resolution of 30´´ grid. These airline distances were assigned correspondently to each sample.

Fish were sampled by active fishing methods with the help of local fishermen who could use the fishing gear in various habitats. Two fishermen used a 1.25 cm mesh-size cast-nets and two others used a 1 cm mesh-size sweep-net (1.5 × 4 m). At each site, fish were collected on river-turns, banks with submerged vegetation, woody debris accumulation, and shadowed areas for half hour per site. To capture fish in rocky reaches with fast flowing waters, a cast-net was swept by two fishermen for two minutes along the current.

All individual fish were weighed (net weight – NW), and their standard length (SL) were measured immediately after the sampling. Each fish sample was dissected, and samples of the same species were placed in polyethylene bags, labeled, and frozen until delivery at the Centro de Estudios Aplicados en Química (CESAQ-PUCE) laboratory for THg determination in fish muscle.

All fish were classified by their dietary habits (qualitative variable): omnivorous, periphyton-feeder, and carnivorous. Three fish species were considered as omnivorous (*Bryconamericus dahli*, *Brycon* sp.*,* and *Brycon dentex*), *Chaetostoma marginatum* [51,56] was considered as a periphyton-feeder; and there were four carnivorous species (*Pimelodella modestus*, *Rhamdia quelen*, *Gobiomorus maculatus, Mesoheros festae* [48,51,57,58]).

Ethical approval was not required for this project since no analyses were performed on living specimens. The wild fish collection and access to the field sites were carried out under research permit N°016–2019-IC-FAU-DPE-MA issued by the Ministerio de Ambiente y Energía del Ecuador. No other permit was required.

### Sample preparation and analysis

At the laboratory, each individual fish was washed with high-quality reagent water (resistivity of 18.2 MΩ·cm at 25 °C) then immediately frozen and stored at −25 °C. Fish samples were freeze-dried for 48 hours using a freeze-dryer (LaboGene, CoolSafe, Denmark) at −50 °C and 0.150 hPa until a constant weight was obtained. The loss in weight was calculated as the percentage of water content [59–62]. This pre-treatment is also necessary to obtain dry samples for further acid digestion. Each dry fish individual was ground with a porcelain mortar and pestle and then transferred to a plastic bag. Appropriate washing procedures were performed to avoid cross-contamination between samples during the grinding step. After grinding, some samples of the same species from each location with same length and body weight were pooled in pairs, homogenized, and stored in a desiccator until the acid digestion procedure. From a total of 307 individuals (S1 Table), 142 pooled samples were obtained and analyzed.

Total Hg content of fish tissues was analyzed following the method described by Yánez-Jácome et al. [63]. Each dried sample was weighed (0.3 g) in a high-pressure polytetrafluoroethylene vessel, MARS EasyPrep, and solubilized with 1 mL of HNO_3_ (trace metal grade, Certified ACS, Fisher Chemical, Canada, CAS# 7697–37–2), 1 mL of H_2_O_2_ (Certified ACS, Fisher Chemical, Canada, CAS# 7722–84–1) and 1 mL of HClO_4_ (Certified ACS, Fisher Chemical, Canada, CAS# 7601–90–3) using a microwave digestion system (CEM, MARS 6, USA). Samples were gently mixed and vials were left open for 10 min before closing them. The microwave parameters were: 100% power (1400 W), pressure at 800 psi, temperature was increased to 210 °C over 20 min and maintained at 210 °C for a further 15 min. After cooling, the solution was filtered at <20 µm and diluted up to 50 mL. One milliliter of HCl (Certified ACS, Fisher Chemical, Canada, CAS# 7647-01-0) was added to each digested sample to reach a final concentration of HCl (2%).

The samples were analyzed by cold vapor atomic fluorescence spectrometry (Analytik Jena, Mercur Plus, Germany). The detection and quantification limits of THg in fish were 0.003 µg.g^-1^ and 0.012 µg.g^-1^ on a dry weight basis (d.w.), respectively. A mercury external standard multipoint calibration curve was prepared with appropriate dilution of Hg certified stock solution (9.995 ± 0.056 µg.L^-1^; NIST-traceable, Inorganic Ventures, USA). The coefficient of determination R^2^ was ≥ 0.99. For quality assurance/quality control sets, a certified reference material (CRM) DORM-4 (fish protein) from the National Research Council of Canada was analyzed. The mean measured THg concentrations in the CRM was 0.389 ± 0.023 µg.g^-1^ (d.w.) corresponding to a mean recovery of 94.5 ± 0.056% (n = 26). For each river, fish species were randomly selected and digested in triplicates. The relative standard deviation (RSD) for sample replicates ranged from 2.1% to 8.9% (mean 5.3%, *n* = 259). THg values from analyzed samples in dry weight (d.w.) were converted to wet weight basis (w.w.) considering the loss of water content (between 63.9% and 89.8%) of muscle tissue during the freeze-drying process [59]. THg content is presented in micrograms per gram (µg.g^-1^ w.w.).

### THg exposure assessment

Human exposure due to fish consumption was assessed through the estimated weekly intake (EWI). Assuming a gastric and intestinal bioavailability of 100% for Hg present in fish muscle, the EWI was determined according to Eq. 1:


$$EWI=\frac{\left(C\times IR\right)}{BW}$$ (1)

An average mean body weight (BW) of 14.5 kg, 60 kg, and 70 kg was assumed for children, women, and men, respectively. C is the concentration of THg measured in fish (µg.g^-1^ w.w.); and IR is the weekly ingestion rate (kg.week^-1^). EWI is reported as µg.g^-1^ week.kg of BW^-1^ [60,64].

According to Ormaza et al. [65], annual fish ingestion in Ecuador is under the global average of 21.9 kg per capita, with an annual consumption rate of 10.4 kg per person in the province of Esmeraldas. Therefore, for this study we assumed an optimal weekly ingestion rate of 200 g and 100 g of fish for adults and children, respectively, assuming that children ingest half of adult’s fish portion.

In addition, to emphasize that long-term exposure is important (for contaminants that accumulate in the body), the calculated EWI was compared to the provisional tolerable weekly intake (PTWI) of 1.6 μg.kg^-1^ week⋅kg of BW^-1^ for MeHg proposed by the Joint FAO/WHO Expert Committee on Food Additives [66], to obtain the Margin of Safety (MoS) (Eq. 2).


$$MoS=\frac{EWI}{PTWI}$$ (2)

A value of MoS > 1 indicates there could be adverse effects derived from fish consumption [67,68].

### Statistical analysis

Data are presented as the mean ± standard deviation. As it is known, Hg tends to bioaccumulate along increasing fish size and age, however, according to Johnston et al. (2022) [69], size-standardization with length, weight or age, is not consistent, as specific fish growth rate and food web position may also be linked to variation in mercury concentrations of fish species. Therefore, weight was used as a proxy of the size of the fish since both variables, weight and standard length are highly related (r^2^ = 0.936; *p* < 0.01).

For the present study, the preliminary analysis of the database showed that none of the quantitative variables (fish size, THg content, and airline distance to the river mouth) achieved statistical assumptions to perform parametric statistical analysis, even after performing data transformations. One explanation for this pattern is that some of the variables showed extreme skewness due to fish size variation (and consequently THg concentration). We therefore described general patterns of THg in studied species, considering their size, their feeding habits, sampling sectors, and the distance of sampling sites to the mouth of the main river in the watershed, independently. The found patterns were similar after removing the extreme data outliers; however, they were kept in the dataset because of their importance for the human health risk exposure assessment.

We performed Kruskal Wallis and Paired Wilcoxon tests to assess the differences in THg concentrations between feeding habits and sampling sectors. We also applied a Factorial Analysis of Mixed Data (FAMD), a cluster analysis that facilities insight into the complex relationships between qualitative and quantitative variables. This analysis creates a simplified spatial plane of two main components (represented in axis x and y) where each sample is plotted according to the values on the variables to which it belongs. The classification of the samples according to their categorical variables may provide insights into the importance of the feeding habits or sampling sectors. R software [55] was used to analyze data and to plot results.

## Results and discussion

The 142 analyzed samples belonging to 8 species were used as bioindicators of the environmental impacts of the ASGM in the Northwestern Ecuador, as shown in Supplementary Data (S2 Table). After pooling samples for analysis, according to feeding habits category, 50 samples belong to carnivorous fish, 67 to omnivorous fish, and 25 to periphyton-feeders (S2 Table). Twenty-five fish were caught in the upstream sector, whereas 29, 78 and 10 were caught in the ASGM, abandoned mine and downstream sectors, respectively (S2 Table).

### THg variability in fish samples

Regarding the fish size distribution, *Brycon dentex* and *Rhamdia quelen* were the biggest fish species caught in the present study. For *B. dentex*, the largest SL was 35.4 cm and NW was 514.0 g, meanwhile for *R. quelen* an average SL of 28.4 ± 5.0 cm and NW of 230.9 ± 98.8 g were recorded (S2 Table).

**Fig 2:**
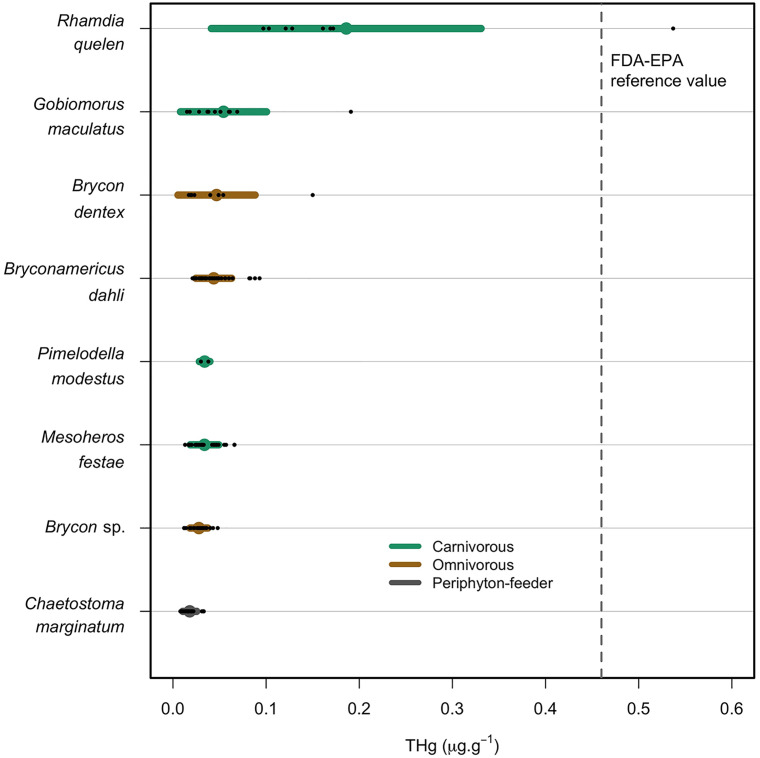
Barplot of total mercury concentrations (THg in µg.g^-1^ w.w.) in fish muscle for different studied species classified by feeding habits. The bar length represents the mean and standard deviation, and the points indicate the distribution of the species samples. Dotted line represents the FDA-EPA reference value for Hg in fish (0.46 µg.g^-1^) [70].

Total Hg mean concentrations in muscle samples varied greatly within fish species, ranging between 0.018 ± 0.023 µg.g^-1^ in *Chaetostoma marginatum* (periphyton-feeder) and 0.186 ± 0.145 µg.g^-1^ in *Rhamdia quelen* (carnivorous). The highest mercury levels were sampled from one individual of *R. quelen* and one of *Gobiomorus maculatus* (carnivorous) at 0.537 µg.g^-1^ and 0.191 µg.g^-1^, respectively (Fig 2). The presence of these outliers skewed the results when analyzing feeding habits and sampling sectors, making their interpretation difficult.

Other studies have found similarly high THg content for *Rhamdia quelen*. Palacios-Torres, et al. (2018) [58] found 0.680 ± 0.10 µg.g^-1^ for an individual with a NW of 221.2 ± 29.8 g and SL of 28.3 ± 1.0 cm, while Salazar-Camacho et al. (2020) [57] reported the highest THg concentration (0.714 µg.g^-1^) for an individual of 24.2 cm. According to Rodríguez Martín-Doimeadios et al. (2014) [71] and Sampaio da Silva et al., (2009) [72] variability of THg among different fish species may be attributed to several factors such as the large range of fish weights, different hydrologic conditions, feeding habits, and land use of the watershed. In the Madeira River, omnivorous fish species have shown a negative and non-statistically significant correlation between size and Hg concentrations [73], demonstrating the complexity of describing generalized metal content related to just one morphological parameter, even more in watersheds perturbated by human activities.

**Fig 3:**
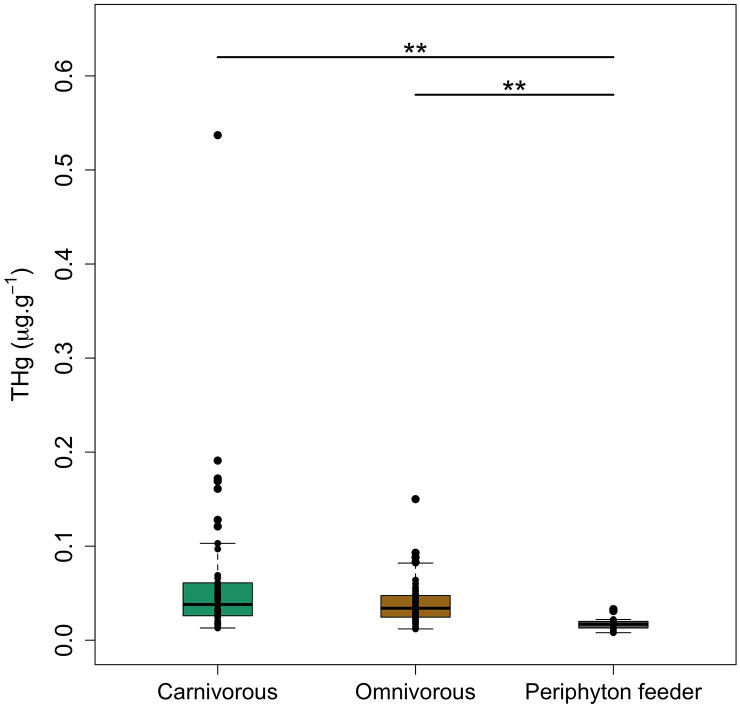
Boxplots of total mercury concentrations (THg in µg.g^-1^ w.w.) in muscles of fish sampled in the study area, by feeding habit. The box length represents the interquartile range, the bar length represents the range, and the horizontal line is the median value. The upper horizontal lines and symbols (**) represent the groups with significative statistical differences (*p* < 0.01).

In relation to feeding habits, we found higher levels of THg in carnivorous fish followed by omnivorous and periphyton-feeder fish (Fig 3; S2 Table). Considering the different diets, THg content in carnivorous fish from Northern Esmeraldas showed almost twice higher concentrations (0.063 ± 0.081 µg.g^-1^) than omnivorous species (0.039 ± 0.022 µg.g^-1^), and over three times the levels of periphyton-feeder species (0.018 ± 0.023 µg Hg·g^-1^). Levels of THg in fish were significantly different in relation to the feeding habits (Kruskal-Wallis chi-squared = 36.362, *p* < 0.01). These differences come from periphyton-feeders being significantly different to the other two categories (*p* < 0.01). These results were expected, however the THg concentrations in carnivorous fish were likely heavily influenced by the presence of the largest individual in that category. Apart from *Rhamdia quelen*, the different species demonstrated similar THg concentrations, despite their feeding habit (Fig 2). The highest THg levels (0.537 µg.g^-1^) were found in the carnivorous fish species (*R. quelen*), while *Chaetostoma. marginatum* (periphyton-feeder) showed the lowest THg concentration (0.008 µg.g^-1^).

Maurice-Bourgoin et al., (2000) [30] reported Hg values between 0.014 and 0.018 µg.g^-1^ in mud feeder species in areas affected by gold mining in South America. Similar results have been shown by Paz-Suconota et al., (2024) [74] in *Chaetostoma* sp*.* collected in the middle basin of the Pastaza River in Morona Santiago province, Ecuador (0.017 ± 0.002 µg.g^-1^). These results are consistent with the present study regarding the periphyton-feeder species (*C. marginatum*).

**Fig 4:**
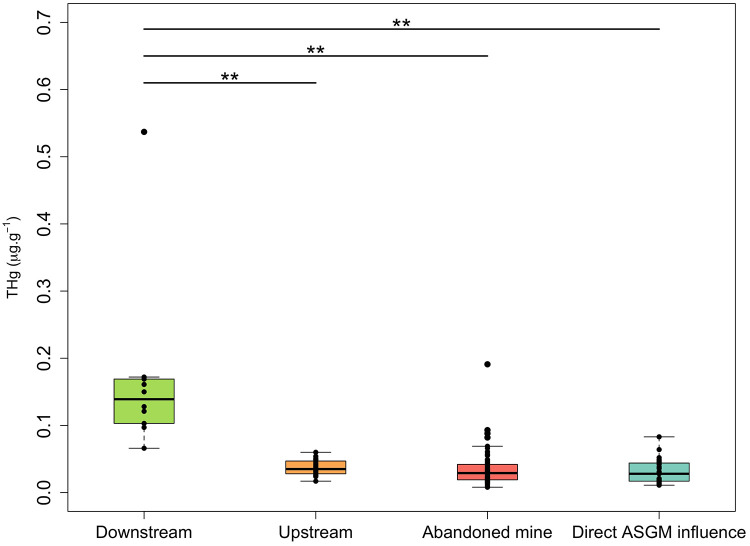
Boxplots of total mercury concentrations (THg in µg.g^-1^ w.w.) in muscles of fish sampled in the study area according to sampling sectors. The box length represents the interquartile range, the bar length represents the range, and the horizontal line is the median value. The upper horizontal lines and symbols (**) represent the groups with significative statistical differences (*p* < 0.01).

Overall, THg levels were found to be significantly different between sampling sectors (Kruskal-Wallis chi-squared = 30.915, *p* < 0.01). While THg concentrations from upstream (0.037 ± 0.011 µg.g^-1^), abandoned mine (0.034 ± 0.026 µg.g^-1^), and direct ASGM influence sampling sectors (0.031 ± 0.018 µg.g^-1^) were similar (Fig 4; S2 Table). THg concentrations in the downstream sector were significantly different to all three other sectors (*p* < 0.01). This pattern is similar even if we analyze data per feeding category (S1 Fig). Downstream sector showed the highest mean THg (0.170 ± 0.133 µg.g^-1^), as the largest individual of the present study corresponds to this category. Surprisingly, we found the direct ASGM influenced sector presented the lowest average of THg.

Our current findings are consistent with previous results from a study performed in the Oyapock Basin of French Guiana where, in upstream areas of ASGM activities, piscivorous fish samples did not demonstrate significantly higher Hg concentrations (2.0 ± 0.9 µg.g^-1^ upstream and 2.9 ± 1.5 µg.g^-1^ in areas affected by ASGM). However, in the same study, THg concentrations of mud feeder species were significantly higher (0.20 ± 0.04 µg.g^-1^) in gold mining areas compared to upstream sites (0.13 ± 0.05 µg.g^-1^) [75]. These findings are contrary to our current results where THg concentrations in *Chaetostoma marginatum* were 0.017 ± 0.003 µg.g^-1^ in the direct ASGM sector, and 0.032 ± 0.001 µg.g^-1^ in the upstream sector. Mud feeder species of periphyton fish exclusively eat biofilm settled on immerged rocks, adventive roots or plant residues. These are important entry points for MeHg due to microbial activity in tropical ecosystems and differences between studies may be attributed to a different composition of the biofilm between the sites [75].

**Fig 5:**
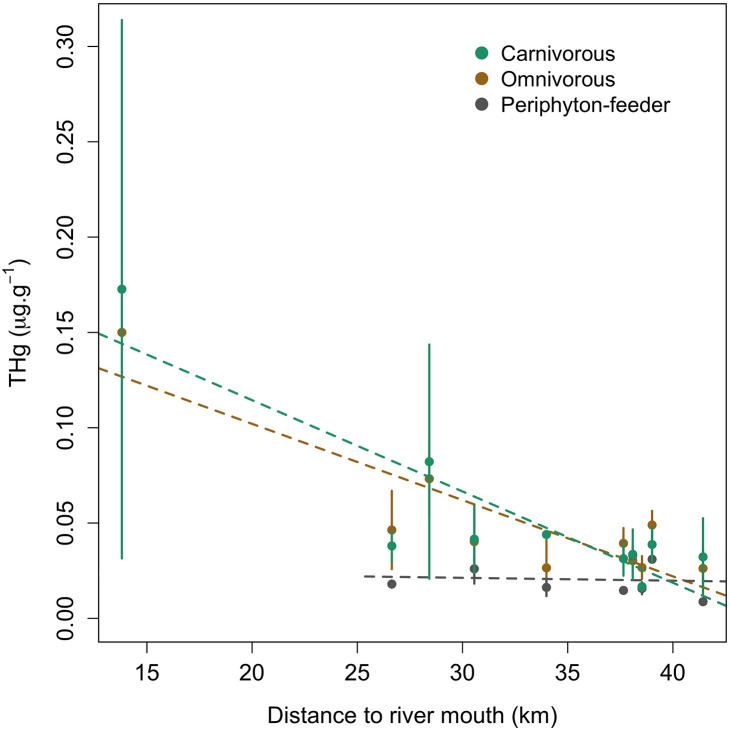
Total mercury, mean and standard deviation for fish pooled by sampling site and feeding habit according to their distance to the mouth of Cayapas River. Dashed lines show the trend in data.

We complemented this analysis by comparing the THg concentrations found in each sampling site with the distance to the watershed main river mouth. The found trend shows that sites closer to the Cayapas River mouth present higher levels of THg (S2 Fig). This pattern is shaped mainly by the THg levels in omnivorous and carnivorous fish (Fig 5). Total mercury levels in periphyton- feeders seem to be low but stable despite the site and sector where they were collected. Higher levels of THg close to the river’s deltas could be attributed to the fact that other human activities can also induce new Hg sources and fluxes in the mainstream, such as agriculture or urban development [76]. Moreover, the occurrence of soil erosion can increase the bioavailability and/or production of MeHg in hydro-systems [35,72], as soil erosion and leaching facilitate transfer of Hg into aquatic ecosystems [77,78]. Additionally, Rebolledo Monsalve et al., (2022) [51] have reported that anthropogenic activities such as deforestation and agriculture are also potential sources of trace metals in the Santiago-Cayapas watershed. All these factors may increase the levels of Hg while the rivers run downstream, diminishing the effect of direct mining activities in specific locations.

### THg concentrations in relation to fish size, feeding habits and sites

**Fig 6:**
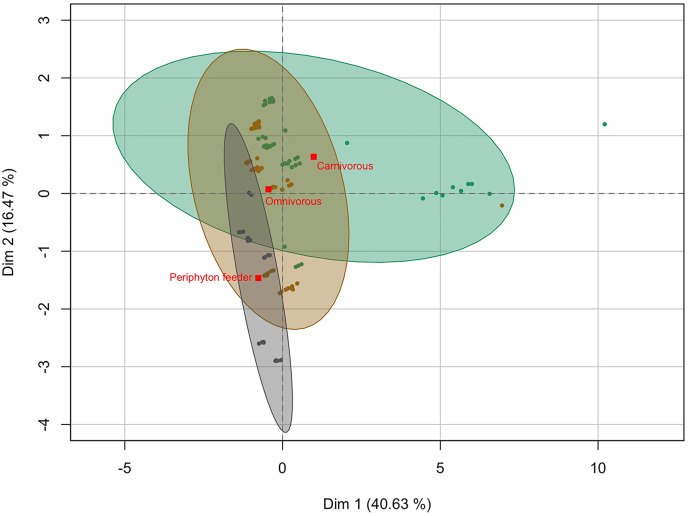
Factorial analysis of mixed data of studied fish samples sorted by feeding habits. Red squares represent the centroid of distribution of the samples for each category and the ellipses around them show a 95% of confidence on the distribution of samples.

According to the FAMD, our results explain 57.10% of the variation in the data set, implying the complexity of finding direct effects between the studied variables (S3 Table). The first component (Dimension 1) represents 40.63% of the variation and it is mainly explained by sampling sector and size variables, and the second component (Dimension 2) represents 16.47% of the variation explained by sampling sector and feeding habits (Fig 6; S3 Table). When we analyze the results according to feeding habits, carnivorous fish occupy a wider space in the graph distributed mainly over the first component axis. The results show an important overlap between samples from the three feeding habits, showing that fish can have similar characteristics as THg concentrations, independently of their trophic level (Fig 6). The results suggest that some carnivorous fish (possibly because of their size and their level in the trophic chain) and some periphyton-feeders (possibly because of their dietary habit) differentiate from the rest of the individuals.

Previous studies in the Amazon basin found differences between the THg content at higher trophic levels compared with the lowest. In the Tapajós Region, one of the areas most threatened by illegal mining in the Brazilian Amazon, Faial et al., (2015) [79] detected much higher mean THg levels in carnivorous species (0.66 µg.g^-1^) than in non-carnivorous (0.09 µg.g^-1^). Moreover, in the same region, higher THg concentrations were found in piscivorous (0.44 µg.g^-1^) than non-piscivorous fish (0.10 µg.g^-1^) [80].

In our study, *Gobiomorus maculatus* and *Rhamdia quelen* demonstrated consistency with the literature data, showing that THg concentration increases at the upper trophic levels of the aquatic food chain [35,56,81]. Likewise, in the study performed by Salazar-Camacho et al., (2020) [57], THg concentrations were quantified in *R. quelen* in five areas of the Atrato River Basin impacted by gold mining activities in the Colombian Pacific Region. The highest Hg concentrations were 1.161 µg.g^-1^ and 0.905 µg.g^-1^, from the Medio Atrato zone and Murindó & Vigía del Fuerte zone, respectively. These authors demonstrated that THg concentrations were not only related to trophic level but also to the total length of the fish. These characteristics have been demonstrated by several authors [59,82,83], showing that biomagnification of Hg takes place in the food chain as a function of the fish size. However, Diringer et al. (2015) [84] and Martinez et al., (2018) [85] reported the contrary, indicating that Hg concentrations were not strongly correlated with length in fish from ASGM areas in Madre de Dios watershed, Peru. Our results are ambiguous since some levels of THg may respond to the size of the fish species as discussed above, but we also found that some fish samples may have similar levels of THg despite their feeding habits (Figs 3 and 6).

THg concentrations were low in periphyton-feeder species (*Chaetostoma marginatum*) suggesting a low content and bioavailability of Hg in water or sediments. A previous study performed in River Idrijca (Slovenia), which is highly contaminated with Hg due to past mercury mining, demonstrated that periphyton is too complex and unpredictable to be used as an indicator of mercury concentrations [86]. Moreover, Lino et al., (2019) [87] reported that biotic and abiotic factors interact in a complex way in the aquatic ecosystem resulting in variable Hg concentrations in a food web.

**Fig 7:**
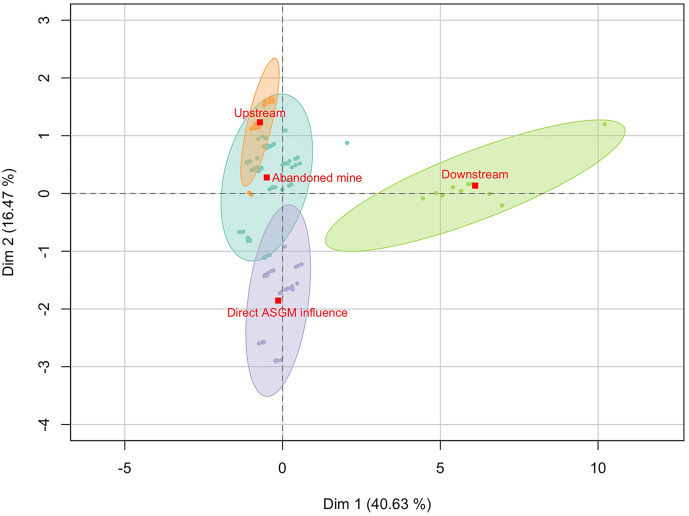
Factorial analysis of mixed data of studied fish samples sorted by sampling sector. Red squares represent the centroid of distribution of the samples for each category and the ellipses around them show a 95% of confidence in the distribution of samples.

Our analysis concerning sampling sector shows an evident difference between downstream samples over the first component. This differentiation is supported by the presence of the largest individuals with high levels of THg and the short distance to the Cayapas River mouth. The other three sampling sectors overlap over the first component, but there is some dispersion over the second component (Fig 7). Direct ASGM sites differentiate from others possibly because of the influence of periphyton-feeder and omnivorous fish samples caught in this sector. *Rhamdia quelen* (the carnivorous fish sample with the highest THg value found) was only captured downstream of the Santiago River, showing consistency with the results of Kasper et al., (2012) [88], who found higher levels of Hg in carnivorous fish in downstream sites from a reservoir in the Amazon region, due to the variability of the prey intake. Predators may present higher levels of Hg due to bioaccumulation than primary consumers [89], which is consistent with the present results.

As reported by Sampaio da Silva et al. (2009) [72], surfaces without forest cover due to deforestation or agricultural practices are also a determining factor directly associated with higher Hg levels in fish due to an increase in sediment load, positively contributing to the presence of particulate Hg in aquatic ecosystems. Mestanza-Ramón et al., (2021, 2022) [50,90] has already reported that indiscriminate deforestation is taking place in Ecuador’s littoral region from mining activities. Illegal gold mining has changed the geographic landscape due to associated soil degradation and erosion which affects approximately 57% of the original forest in the area [50,89].

With illegal gold mining activities causing Hg emissions in the province of Esmeraldas over several decades, it would seem logical that water resources, aquatic and terrestrial ecosystems were negatively impacted [50]. However, our results do not show any significant increases in Hg distribution in fish in current or abandoned mining sectors. According to Maurice-Bourgoin et al., (2000) [35], Hg emitted by the gold extraction activities in the Madeira River headwaters did not directly contaminate the exploited rivers since the Hg is adsorbed in fine particles of suspended matter and transported with the current. This Hg therefore affects the drainage basin waters downstream and is trapped in sediment depository areas and floodplain systems [33,35,91].

The distribution of Hg concentrations in ASGM areas is spatially limited in tropical freshwater systems affected by watershed characteristics, hydrology, sediment dynamics, presence of dams, and specific biogeochemistry of each water body, directly influencing the spatiotemporal patterns of suspended particulate matter (SPM) fluxes and, consequently, SPM-bound Hg fluxes [92,93]. Escobar-Camacho et al., (2024) [94] found that Hg levels in fish tissues from the Piedmont Ecuadorian Amazon, belonging to diverse habitats, species, trophic level, and sizes, cannot be related to the presence or absence of mining activities, which is consistent with our findings.

Our results demonstrate the complexity of understanding the exposure levels in the fish community in the Santiago-Cayapas watershed. To obtain more information about the presence of Hg in the aquatic environment, further ecological and physiological studies on the sampled fish species coupled to a monitoring of mercury contents in the aquatic system (water column, suspended particles and sediments) including spatial and seasonal distribution and progression of the extractive activities in the watershed, should be performed. Such work could assess and modulate the impacts of the ASGM in the Santiago-Cayapas basin compared with other land uses that contribute to the Hg inputs, bioaccumulation, and biomagnification in the ichthyofauna. It is fundamental to provide more site-specific measurements of relative Hg exposure and uptake modes by fauna.

### THg exposure through fish intake

Esmeraldas is considered one of the most excluded and poorest regions in Ecuador [50]. The communities living near the rivers of the Cayapas-Santiago watershed depend on fishing for survival [51]. Freshwater fish constitute their main protein source depending on their availability which varies with the hydrological seasons. Unfortunately, it is well known that fish consumption is the main pathway for MeHg exposure which poses a significant human health risk [83,95,96]. MeHg is a neurotoxin with a lipophilic property [97,98] with harmful effects to multiple important human body systems [99,100]. This toxin can cross the blood-brain barriers or placenta, concentrating in the fetus and his/her central nervous system, causing potentially adverse neurological effects and developmental disorders [98]. Care should be taken, especially with vulnerable groups, such as pregnant women and children [14,101].

The only fish sample from this study to exceed the FDA-EPA reference value of 0.46 µg.g^-1^ [70] was from the species *Rhamdia quelen* (0.537 µg.g^-1^) which was caught in the downstream sector. The tolerable weekly intake of fish and MoS for the different fish species consumption is presented in Table 1. All results were <1, showing no adverse effects derived from fish consumption.

**Table 1:** THg measured average concentration per study fish species, tolerable weekly intake of fish meat (in kg) for men, women and children, and Margin of Safety (MoS) for one weekly fish intake of 200 g and 100 g for adults and children, respectively.

Fish species	THg average concentration (µg.g^-1^ w.w.)	Recommended weekly intake (kg fish/weekly)			MoS		
		Children^a^	Women^b^	Men^c^	Children	Women	Men
***Brycon dentex***	0.047	0.50	2.06	2.41	0.20	0.10	0.08
***Brycon* sp.**	0.028	0.83	3.44	4.01	0.12	0.06	0.05
***Bryconamericus dahli***	0.044	0.53	2.19	2.56	0.19	0.09	0.08
***Chaetostoma marginatum***	0.018	1.30	5.36	6.26	0.04	0.03	0.08
***Gobiomorus maculatus***	0.054	0.43	1.77	2.06	0.23	0.11	0.10
***Mesoheros festae***	0.034	0.69	2.83	3.31	0.15	0.07	0.06
***Pimelodella modestus***	0.034	0.69	2.84	3.31	0.15	0.07	0.06
***Rhamdia quelen***	0.186	0.12	0.52	0.60	0.80	0.39	0.33

^a^Body weight of 14.5 kg for children; ^b^ Body weight of 60 kg for women; ^c^ Body weight of 70 kg for men.

PTWI (µg MeHg.kg^-1^ HBW) = 1.6.

According to the FDA/EPA recommendations, fish (with a maximum allowable average mercury concentration of 0.15 µg.g^-1^) should only be consumed by adults and children up to 2 and 3 servings per week at 113 g and 57 g, respectively [70].

Human communities which are dependent on fish for food consume a higher weekly ingestion rate (800 g and 400 g per week for adults and children, respectively) which is approximately 7 times the FDA/EPA serving size recommendations. When this ingestion rate is applied to our study, the MoS exceeds 1 for *Rhamdia quelen*, therefore posing a potential human health risk from fish consumption in the three population groups. On the other hand, with a fish intake of 2 and 3 times per week for adults and children, respectively, no significant potential health risk was evident. However, special attention should be focused on carnivorous fish from the downstream sector, as higher MoS (0.91 and 1.37) were found for children (Supplementary Data, S4 Table), due to the occurrence of THg in higher trophic levels or large fish making fish unsuitable for human consumption [48].

Alternate studies of Hg levels in fish potentially linked to gold mining areas in the Amazon region of Ecuador have demonstrated that these levels can lead to progressive bioaccumulation and increase the risk of chronic health problems, especially in those communities dependent on fish as a main protein source. While some fish species from the middle basin of the Pastaza River did not exceed the national maximum permissible limits (1 µg.g^-1^) [74,102], fish from the Northern Ecuadorian Amazon and the Napo Basin exhibited high Hg concentrations underscoring the need for risk assessments of Hg exposure in Amazonian communities [94,103]. In other Latin American regions, studies have shown that elevated fish consumption rates in riparian communities amplify their vulnerability to Hg exposure risks. It is therefore necessary to identify fish species that have lower mercury levels and design dietary strategies that mitigate risk to the community’s health [104–107].

The THg concentrations in fish and the human health risk assessment presented in this study cannot be directly correlated to the contamination risk of the area by ASGM activities. This is the first approach to evaluate the THg concentrations in fish consumed by Northern Esmeraldas population. It might be important to perform biomonitoring analyzes, where THg levels are quantified in human hair from people living in communities of the Northern Esmeraldas region, mainly in pregnant women and children —the most vulnerable part of the population— to assess the actual accumulated levels of THg considering their dietary behaviors (serving size, type and origin of consumed fish), and long-term exposure.

## Conclusions

Findings from our exploratory study demonstrate that the average Hg bioaccumulation in fish was not uniformly distributed among species, feeding habits, and the four different sampling sectors in the Santiago-Cayapas watershed. The results show that the understanding of the THg pollution is complex and further investigations are required.

The THg varied within all three feeding habit categories. The THg level in carnivorous fish was higher than those in omnivorous and periphyton- feeder fish, which can be related to the fact that the carnivorous fish have higher fish size including the largest individual in the study. Samples from sites under different levels of influence of mining activities, may have similar THg content, suggesting that the THg in the fish tissues is independent of direct mining activities for the studied species here. Fish in the downstream sector showed higher levels of Hg maybe due to biomagnification and presence of larger fish in the area, as well as other sources of Hg from human activities contributing to additional Hg inputs along the river.

Higher levels of THg were found closer to the mouth of the Cayapas River which is mainly due to the levels of THg in carnivorous and omnivorous fish samples. The extreme levels of THg in the downstream sector are also influenced by the finding of large fish in the zone.

Our investigation regarding the THg exposure through fish intake suggested that the fish species sampled were safe for human consumption if ingestion rates are limited to 2–3 servings per week, according to the U.S. EPA recommendations. However, only one individual fish of the carnivorous species *Rhamdia quelen* exceeded the FDA-EPA Hg reference value indicating that THg content in higher trophic levels can threaten human health, especially populations which are exclusively dependent on fish as a protein source.

## Supporting information

S1 Table Fish individuals (*n* = 307) captured in water bodies from the Santiago-Cayapas Basin. (DOCX)

S2 Table Mean THg concentrations (THg, µg.g^-1^ w.w. ) by feeding habits, number of samples (*n*), standard length range, net weight, for the 142 fish pooled samples obtained from the Santiago-Cayapas watershed in Esmeraldas province, Ecuador.
(DOCX)

S3 Table Factorial Analysis of Mixed Data (FAMD) eigen values and contribution of each variable to each dimension. (DOCX)

S4 Table Mercury levels in muscle tissue of fish species under study, estimated weekly intake for children, women, and men, based on a weekly ingestion rate of 57 g fish.day^-1^ and 113 g fish.day^-1^ for children and adults, respectively. (DOCX)

S1 Fig Boxplot of THg concentrations in sampling sectors by feeding habits. (TIF)

S2 Fig Mean THg concentrations by sampling sites according to distance to Cayapas River mouth. Bars represent standard deviations. Dashed line represents the trend in data.
(TIF)
