# Supplementary material for: Total mercury contamination in fish species of Northwestern Ecuador and potential human health risks
Source: PLoS One. 2026 Feb 19;21(2):e0342455. doi: 10.1371/journal.pone.0342455 (PMC12919828; doi:10.1371/journal.pone.0342455)
Supplement: S1 Table — (DOCX) [file pone.0342455.s001.docx]

**S1 Table. Fish individuals (n=307) captured in water bodies from the Santiago-Cayapas Basin.**

| River / Estuary | Site | Latitude | Longitude | Distance to Cayapas river mouth (km) | Sector | Fish specie | Feeding habits | Samples collected | Total of samples per  water body |
| --- | --- | --- | --- | --- | --- | --- | --- | --- | --- |
| Comba | Comba | 1,01101 | -78,68241 | 39.01 | Upstream site | ^1^*Brycon dentex* | O | 9 | 30 |
|  |  |  |  |  |  | ^2^*Bryconamericus dahli* | O | 10 |  |
|  |  |  |  |  |  | ^3^*Chaetostoma marginatum* | P | 1 |  |
|  |  |  |  |  |  | ^4^*Mesoheros festae* | C | 10 |  |
| Quebrada del Parto | Quebrada del Parto | 0,84016 | -78,87319 | 38.09 | Upstream site | ^1^*Brycon* sp. | O | 11 | 29 |
|  |  |  |  |  |  | ^2^*Bryconamericus dahli* | O | 11 |  |
|  |  |  |  |  |  | ^3^*Chaetostoma marginatum* | P | 1 |  |
|  |  |  |  |  |  | ^5^*Gobiomorus maculatus* | C | 4 |  |
|  |  |  |  |  |  | ^4^*Mesoheros festae* | C | 2 |  |
| Tululbí | Minas Viejas | 1,08423 | -78,63552 | 41.43 | Abandoned mine | ^1^*Brycon dentex* | O | 10 | 38 |
|  |  |  |  |  |  | *^2^Bryconamericus dahli* | O | 10 |  |
|  |  |  |  |  |  | ^3^*Chaetostoma marginatum* | P | 10 |  |
|  |  |  |  |  |  | ^5^*Gobiomorus maculatus* | C | 1 |  |
|  |  |  |  |  |  | ^4^*Mesoheros festae* | C | 7 |  |

| River / Estuary | Site | Latitude | Longitude | Distance to Cayapas river mouth (km) | Sector | Fish specie | Feeding habits | Samples collected | Total of samples per  water body |
| --- | --- | --- | --- | --- | --- | --- | --- | --- | --- |
| Durango | Durango | 1,07149 | -78,66524 | 38.53 | Abandoned mine | ^1^*Brycon* sp. | O | 10 | 40 |
|  |  |  |  |  |  | ^2^*Bryconamericus dahli* | O | 10 |  |
|  |  |  |  |  |  | ^3^*Chaetostoma marginatum* | P | 6 |  |
|  |  |  |  |  |  | ^5^*Gobiomorus maculatus* | C | 2 |  |
|  |  |  |  |  |  | ^4^*Mesoheros festae* | C | 12 |  |
| Uimbicito | Uimbicito | 1,0122 | -78,76802 | 30.56 | Abandoned mine | ^1^*Brycon* sp. | O | 8 | 42 |
|  |  |  |  |  |  | ^1^*Bryconamericus dahli* | O | 11 |  |
|  |  |  |  |  |  | ^3^*Chaetostoma marginatum* | P | 9 |  |
|  |  |  |  |  |  | ^5^*Gobiomorus maculatus* | C | 3 |  |
|  |  |  |  |  |  | ^4^*Mesoheros festae* | C | 11 |  |
| Estero María | María Alto | 0,93141 | -78,88338 | 28.42 | Abandoned mine | ^1^*Bryconamericus dahli* | O | 10 | 21 |
|  |  |  |  |  |  | ^5^*Gobiomorus maculatus* | C | 1 |  |
|  |  |  |  |  |  | ^4^*Mesoheros festae* | C | 10 |  |
| Zapallito | Aquí me quedo | 0,82664 | -78,93135 | 37.65 | Abandoned mine | ^1^*Brycon* sp. | O | 10 | 36 |
|  |  |  |  |  |  | ^2^*Bryconamericus dahli* | O | 9 |  |
|  |  |  |  |  |  | ^3^*Chaetostoma marginatum* | P | 5 |  |
|  |  |  |  |  |  | ^5^*Gobiomorus maculatus* | C | 2 |  |
|  |  |  |  |  |  | ^4^*Mesoheros festae* | C | 10 |  |

| River / Estuary | | Site | Latitude | | Longitude | | Distance to Cayapas river mouth (km) | Sector | Fish specie | | Feeding habits | | Samples collected | | Total of samples per  water body | |
| --- | --- | --- | --- | --- | --- | --- | --- | --- | --- | --- | --- | --- | --- | --- | --- | --- |
| Cachabí | | Los Ajos | 1,02057 | | -78,72794 | | 33.99 | Direct ASGM influence | ^1^*Brycon* sp. | | O | | 10 | | 33 | |
|  |  |  |  |  |  |  |  |  | ^2^*Bryconamericus dahli* | | O | | 12 | |  |  |
|  |  |  |  |  |  |  |  |  | ^3^*Chaetostoma marginatum* | | P | | 10 | |  |  |
|  |  |  |  |  |  |  |  |  | ^4^*Mesoheros festae* | | C | | 1 | |  |  |
| San Javier | San Javier | | 1,06553 | -78,77983 | | 26.64 | | Direct ASGM influence | | ^1^*Brycon* sp. | | O | | 3 | | 27 |
|  |  |  |  |  |  |  |  |  |  | *^2^Bryconamericus dahli* | | O | | 11 | |  |
|  |  |  |  |  |  |  |  |  |  | ^3^*Chaetostoma marginatum* | | P | | 10 | |  |
|  |  |  |  |  |  |  |  |  |  | ^4^*Mesoheros festae* | | C | | 1 | |  |
|  |  |  |  |  |  |  |  |  |  | ^6^*Pimelodella modestus* | | C | | 2 | |  |
| Santiago | Maldonado | | 1,07578 | -78,90841 | | 13.81 | | Downstream site | | ^2^*Brycon dentex* | | O | | 1 | | 11 |
|  |  |  |  |  |  |  |  |  |  | ^4^*Mesoheros festae* | | C | | 1 | |  |
|  |  |  |  |  |  |  |  |  |  | ^6^*Rhamdia quelen* | | C | | 9 | |  |
| C: carnivorous; O: omnivorous; P: periphyton-feeder.  Families: ^1^Bryconidae; ^2^Characidae; ^3^Loricariidae; ^4^Cichlidae; ^5^Eleotridae; ^6^Heptapteridae. | | | | | | | | | | | | | | | | |
