# Supplementary material for: Total mercury contamination in fish species of Northwestern Ecuador and potential human health risks
Source: PLoS One. 2026 Feb 19;21(2):e0342455. doi: 10.1371/journal.pone.0342455 (PMC12919828; doi:10.1371/journal.pone.0342455)
Supplement: S2 Table — ) by feeding habits, number of samples (n), standard length range, net weight, for the 142 fish pooled samples obtained from the Santiago-Cayapas watershed in Esmeraldas province, Ecuador. (DOCX) [file pone.0342455.s002.docx]

**S2 Table. Mean THg concentrations (THg, µg.g^-1^ w.w.) by feeding habits, number of samples (*n*), standard length range, net weight, for the 142 fish pooled samples obtained from the Santiago-Cayapas watershed in Esmeraldas province, Ecuador.**

| Sampling sector | Scientific name | Feeding habits | Sample size | Standard length range (cm) (Average) | Net weight range (g) (Average) | THg concentration range (µg.g^-1^ w.w.)  (Average) |
| --- | --- | --- | --- | --- | --- | --- |
| Upstream site | *Brycon dentex***^a^** | O | 3 | 9.75 – 11.10 (10.45) | 10.17 – 13.44 (12.05) | 0.040 – 0.054  (0.048) |
|  | *Brycon* sp.**^a^** | O | 4 | 7.15 – 14.00 (10.81) | 4.11 – 38.00 (20.27) | 0.026 – 0.032  (0.028) |
|  | *Bryconamericus dahli***^b^** | O | 7 | 5.50 – 8.40 (7.26) | 1.95 – 9.10  (5.53) | 0.024 – 0.060  (0.040) |
|  | *Chaetostoma marginatum***^b^** | P | 2 | 8.70 – 8.80 (8.75) | 7.51 – 8.30 (7.91) | 0.031 – 0.033  (0.032) |
|  | *Gobiomorus maculatus*^a^ | C | 3 | 8.80 – 14.20 (11.13) | 5.97 – 25.00 (13.18) | 0.037 – 0.051  (0.042) |
|  | *Mesoheros festae***^a^** | C | 6 | 5.50 – 9.00 (6.68) | 2.29 – 13.25 (6.04) | 0.017 – 0.049  (0.033) |

| Sampling sector | Scientific name | Feeding habits | Sample size | Standard length range (cm) (Average) | Net weight range (g) (Average) | THg concentration range (µg.g^-1^ w.w.)  (Average) |
| --- | --- | --- | --- | --- | --- | --- |
| Abandoned mining site | *Brycon dentex*^a^ | O | 5 | 7.50 – 14.20  (10.99) | 4.49 – 24.0  (14.64) | 0.017 – 0.049  (0.025) |
|  | *Brycon* sp.^a^ | O | 11 | 5.65 – 15.40 (11.28) | 3.62 – 92.00 (25.93) | 0.022 – 0.043  (0.031) |
|  | *Bryconamericus dahli*^b^ | O | 20 | 5.40 – 10.40 (7.75) | 1.84 – 19.37 (6.90) | 0.021 – 0.093  (0.043) |
|  | *Chaetostoma marginatum***^b^** | P | 14 | 5.75 – 12.30 (8.56) | 2.54 – 21.71 (8.83) | 0.008**^*^** – 0.033 (0.016) |
|  | *Gobiomorus maculatus***^a^** | C | 9 | 7.70 – 21.20 (13.02) | 4.01 – 80.00 (26.31) | 0.015 – 0.191  (0.058) |
|  | *Mesoheros festae***^a^** | C | 19 | 3.55 – 13.10 (6.37) | 0.77 – 37.64 (6.71) | 0.013 – 0.066  (0.031) |

| Sampling sector | Scientific name | Feeding habits | Sample size | Standard length range (cm) (Average) | Net weight range (g) (Average) | THg concentration range (µg.g^-1^ w.w.)  (Average) |
| --- | --- | --- | --- | --- | --- | --- |
| Direct ASGM influence | *Brycon dentex***^a^** | O | 7 | 7.40 – 18.30 (13.19) | 8.02 – 50.00 (23.15) | 0.012 – 0.048  (0.023) |
|  | *Bryconamericus dahli***^b^** | O | 9 | 6.80 – 9.00 (7.86) | 4.05 – 10.57 (6.87) | 0.028 – 0.083  (0.047) |
|  | *Chaetostoma marginatum***^b^** | P | 9 | 5.70 – 18.20  (9.45) | 2.31 – 33.06  (12.78) | 0.011 – 0.021  (0.017) |
|  | *Mesoheros festae***^a^** | C | 2 | 6.20 – 8.20 (7.20) | 3.70 – 9.52  (6.61) | 0.044 – 0.046  (0.045) |
|  | *Pimelodella modestus***^b^** | C | 2 | 7.30 – 8.80 (8.05) | 2.95 – 5.27 (4.11) | 0.030 – 0.038  (0.034) |
| Downstream | *Brycon dentex***^a^** | O | 1 | 35.40 | 514.00 | 0.150 |
|  | *Mesoheros festae***^a^** | C | 1 | 29.80 | 495.00 | 0.066 |
|  | *Rhamdia quelen***^a^** | C | 8 | 21.10 – 37.40 (28.36) | 11.400 – 430.00 (230.88) | 0.097 – 0.537  (0.186) |
| C: carnivorous; O: omnivorous; P: periphyton-feeder. | | | | | | |
