# Supplementary material for: Total mercury contamination in fish species of Northwestern Ecuador and potential human health risks
Source: PLoS One. 2026 Feb 19;21(2):e0342455. doi: 10.1371/journal.pone.0342455 (PMC12919828; doi:10.1371/journal.pone.0342455)
Supplement: S3 Table — (DOCX) [file pone.0342455.s003.docx]

**S3 Table. Factorial Analysis of Mixed Data (FAMD) eigen values and contribution of each variable to each dimension**

| Parameters | First component (Dimension 1) | Second component (Dimension 2) |
| --- | --- | --- |
| Eigen value | 3.2503 | 1.3172 |
| Percentage of variance (%) | 40.63 | 16.47 |
| Fish size (net weight) | 25.02 | 0.11 |
| THg concentration | 20.62 | 1.90 |
| Distance to mouth river | 22.18 | 9.53 |
| Sampling site | 27.05 | 58.39 |
| Feeding habits | 5.13 | 30.07 |
