# Supplementary material for: Total mercury contamination in fish species of Northwestern Ecuador and potential human health risks
Source: PLoS One. 2026 Feb 19;21(2):e0342455. doi: 10.1371/journal.pone.0342455 (PMC12919828; doi:10.1371/journal.pone.0342455)
Supplement: S4 Table — (DOCX) [file pone.0342455.s004.docx]

**S4 Table. Mercury levels in muscle tissue of fish species under study, estimated weekly intake for children, women, and men, based on a weekly ingestion rate of 57 g fish.day^-1^ and 113 g fish.day^-1^ for children and adults, respectively.**

| Fish species | THg average concentration (µg.g^-1^ w.w.) | Children ^a^ | | Women ^b^ | | Men ^c^ | |
| --- | --- | --- | --- | --- | --- | --- | --- |
|  |  | **2 servings /week** | **3 servings /week** | **2 servings /week** | **3 servings /week** | **2 servings /week** | **3 servings /week** |
|  |  | **MoS** | **MoS** | **MoS** | **Mos** | **MoS** | **MoS** |
| *Brycon dentex* | 0.047 | 0.23 | 0.34 | 0.11 | 0.16 | 0.09 | 0.14 |
| *Brycon* sp. | 0.028 | 0.14 | 0.21 | 0.07 | 0.10 | 0.06 | 0.08 |
| *Bryconamericus dahli* | 0.044 | 0.21 | 0.32 | 0.10 | 0.15 | 0.09 | 0.13 |
| *Chaetostoma marginatum* | 0.018 | 0.09 | 0.13 | 0.04 | 0.06 | 0.04 | 0.05 |
| *Gobiomorus maculatus* | 0.054 | 0.27 | 0.40 | 0.13 | 0.19 | 0.11 | 0.16 |
| *Mesoheros festae* | 0.034 | 0.17 | 0.25 | 0.08 | 0.12 | 0.07 | 0.10 |
| *Pimelodella modestus* | 0.034 | 0.17 | 0.25 | 0.08 | 0.12 | 0.07 | 0.10 |
| *Rhamdia quelen* | 0.186 | **0.91** | **1.37** | 0.38 | 0.66 | 0.38 | 0.56 |
| ^a^ Body weight of 14.5 kg for children; ^b^ Body weight of 60 kg for women; ^c^ Body weight of 70 kg for men  PTWI (µg MeHg.kg^-1^ HBW) = 1.6 | | | | | | | |
