# Supplementary figures and images for: Total mercury contamination in fish species of Northwestern Ecuador and potential human health risks
Source: PLoS One. 2026 Feb 19;21(2):e0342455. doi: 10.1371/journal.pone.0342455 (PMC12919828; doi:10.1371/journal.pone.0342455)

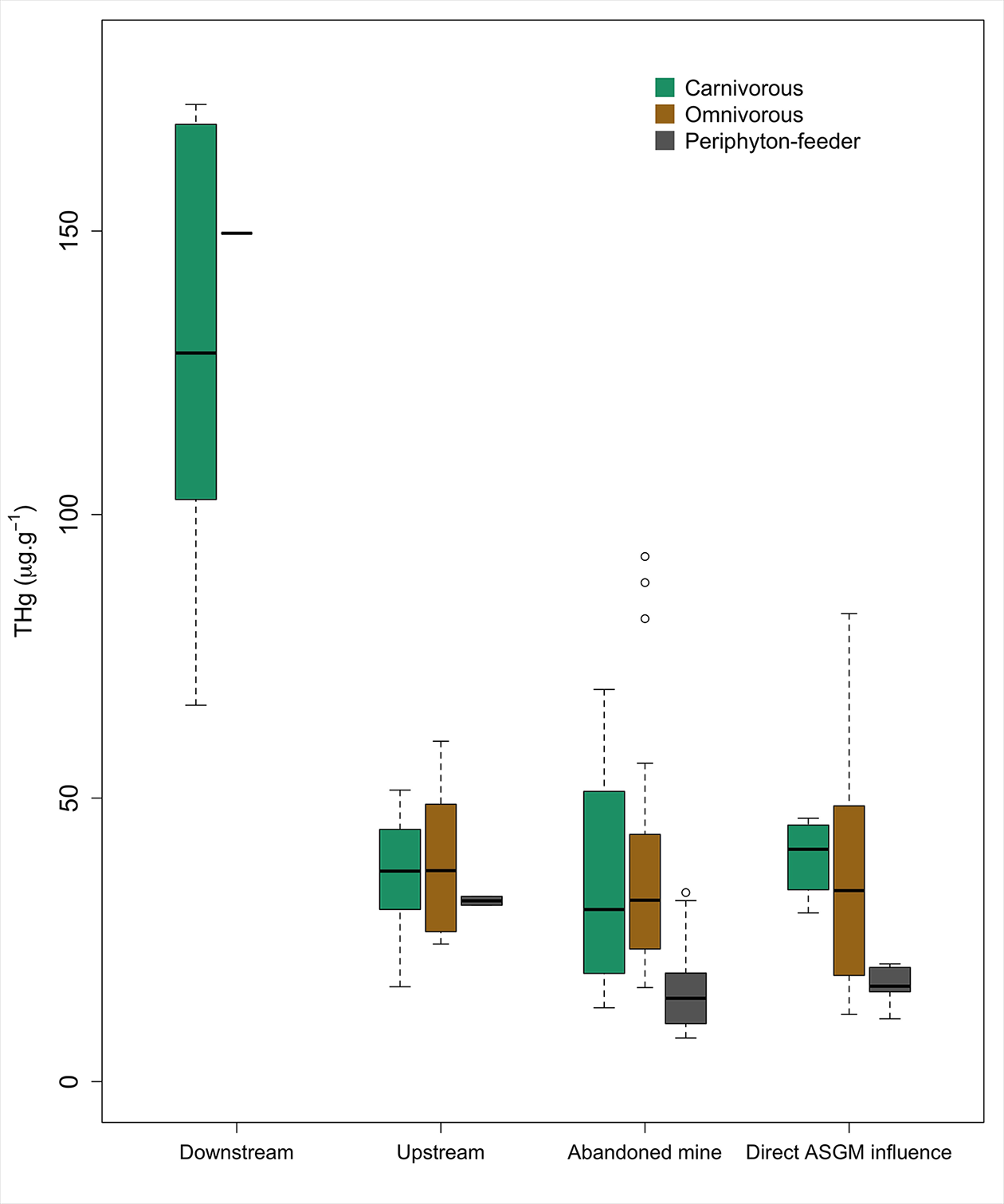

Supplement: S1 Fig — (TIF) [file pone.0342455.s005.tif]

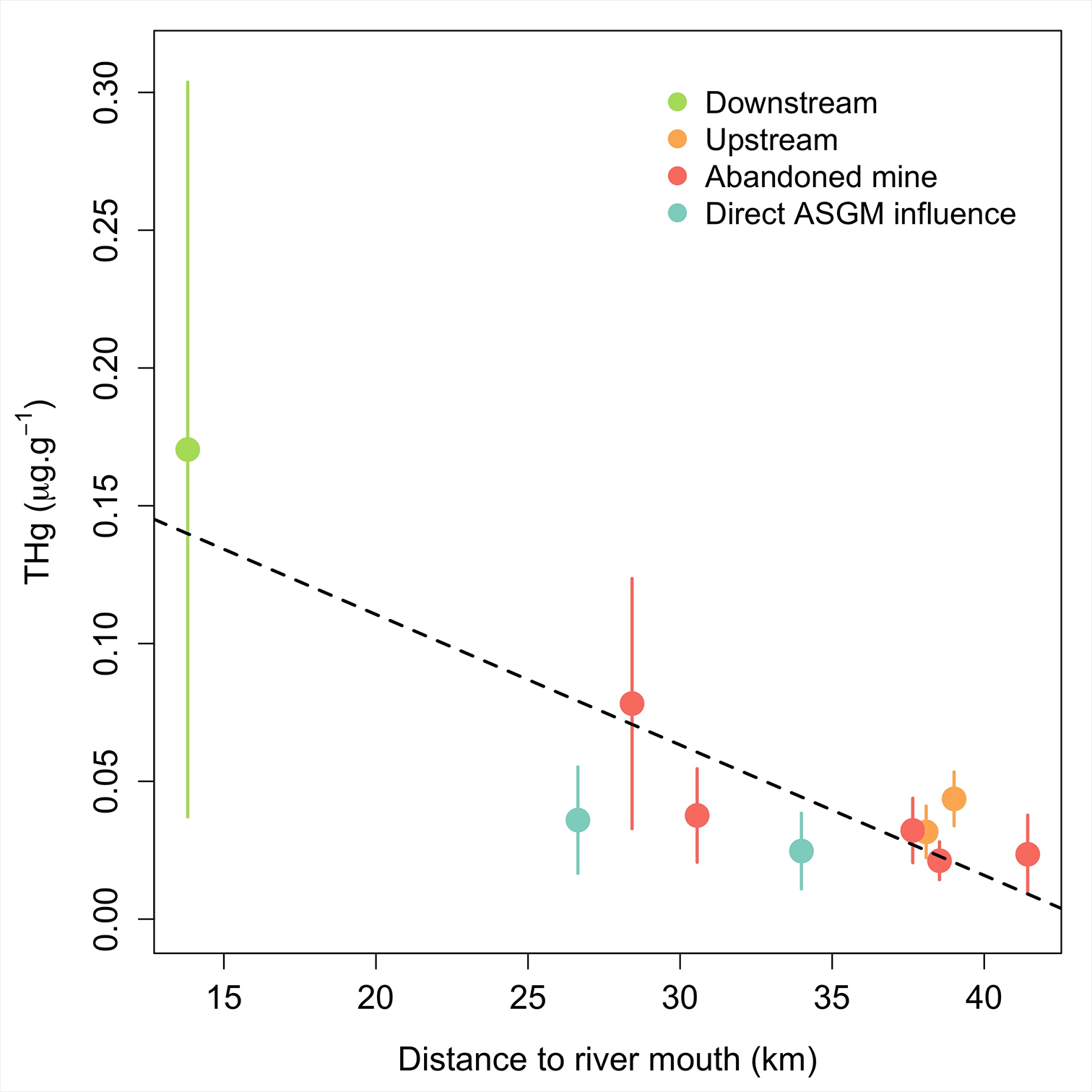

Supplement: S2 Fig — Bars represent standard deviations. Dashed line represents the trend in data. (TIF) [file pone.0342455.s006.tif]
